# Comparative implementation-effectiveness of three strategies to perform hearing screening among older adults in primary care clinics: study design and protocol

**DOI:** 10.1186/s12877-020-01576-x

**Published:** 2020-05-11

**Authors:** Janet Prvu Bettger, Rowena J. Dolor, David L. Witsell, Judy R. Dubno, Carl F. Pieper, Amy R. Walker, Mina Silberberg, Kristine A. Schulz, Pranab Majumder, Erika Juhlin, Sherri L. Smith, Howard W. Francis, Debara L. Tucci

**Affiliations:** 1 Duke University School of Medicine, DUMC Box 104002, Durham, NC 27710 USA; 2 Division of General Internal Medicine, Department of Medicine, Duke University, 200 Morris Street, 3rd floor, Durham, NC 27701 USA; 3 Department of Head and Neck Surgery & Communication Sciences, Duke University Medical Center, DUMC Box 3805, Durham, NC 27710 USA; 4 Department of Otolaryngology-Head and Neck Surgery, Medical University of South Carolina, 135 Rutledge Avenue, MSC 550, Charleston, SC 29425-5500 USA; 5 Center for the Study of Aging and Human Development, Department of Biostatistics and BioInformatics, Duke University School of Medicine, DUMC Box 3003, Durham, NC 27710 USA; 6 Family Medicine and Community Health, Duke University Medical Center, DUMC 104652, Durham, NC 27710 USA; 7 Fuqua School of Business, Duke University, 100 Fuqua Drive, Durham, NC 27708 USA

**Keywords:** Hearing loss, Hearing healthcare, Hearing disorders, Primary care, Older adults, Implementation science, Effectiveness

## Abstract

**Background:**

The burden of hearing loss among older adults could be mitigated with appropriate care. This study compares implementation of three hearing screening strategies in primary care, and examines the reliability and validity of patient self-assessment, primary care providers (PCP) and diagnostic audiologists in the identification of ‘red flag’ conditions (those conditions that may require medical consultation and/or intervention).

**Methods:**

Six primary care practices will implement one of three screening strategies (2 practices per strategy) with 660 patients (220 per strategy) ages 65–75 years with no history of hearing aid use or diagnosis of hearing loss. Strategies differ on the location and use of PCP encouragement to complete a telephone-based hearing screen (tele-HS). Group 1: instructions for tele-HS to complete at home and educational materials on warning signs and consequences of hearing loss. Group 2: PCP counseling/encouragement on importance of hearing screening, instructions to take the tele-HS from home, educational materials. Group 3: PCP counseling/encouragement, in-office tele-HS, and educational materials. Patients from all groups who fail the tele-HS will be referred for diagnostic audiological testing and medical evaluation, and complete a self-assessment of red flag conditions at this follow-up appointment. Due to the expected low incidence of ear disease in the PCP cohort, we will enroll a complementary population of patients (*N* = 500) from selected otolaryngology head and neck surgery clinics in a national practice-based research network to increase the likelihood of occurrence of medical conditions that might contraindicate hearing aid fitting. The primary outcome is the proportion of patients who complete the tele-HS within 2 months of the PCP appointment comparing Group 3 (PCP encouragement, in-office tele-HS, education) versus Groups 2 and 1 (education and tele-HS at home, with and without PCP encouragement, respectively). The several secondary outcomes include direct and indirect costs, patient, family and provider attitudes of hearing healthcare, and accuracy of red flag condition evaluations compared with expert medical assessment by an otolaryngology provider.

**Discussion:**

Determining the relative effectiveness of three different strategies for hearing screening in primary care and the assessment accuracy of red flag conditions can each lead to practice and policy changes that will reduce individual, family and societal burden from hearing loss among older adults.

**Trial registration:**

Clinicaltrials.gov: NCT02928107; 10/10/2016 protocol version 1.

## Background

More than half a billion people worldwide are living with disabling hearing loss and prevalence increases with age [1]. One in three people over age 60 and two thirds of those older than age 70 have hearing loss [2]. Hearing problems can make it difficult to respond to safety warnings, hear and follow healthcare provider advice, and communicate effectively with family and friends. Hearing loss can lead to social isolation with associated increase in the risk of falls, hospitalization, cognitive decline, and poor self-reported health [3–6]. The negative impact is also measurable among spouses of individuals with hearing loss. Being a spouse of someone with hearing impairment increases the likelihood of worse physical and psychological health and social well-being [7, 8]. Effects on the family may not surface until problems manifest in other life areas and hardship ensues.

The financial burden of hearing loss is underestimated because screening for hearing loss is under-utilized resulting in an undetermined number of people affected. Financial burden at the individual level results from lost earnings, low productivity, the inability to secure and maintain employment, the need for premature retirement, and disability costs associated with hearing loss [9–12]. Financial burden is compounded at the family level with an unmeasured and uncertain amount of informal care currently provided or projected to be needed by family and others. Further, there are societal costs for communication difficulties, social isolation, stigma, healthcare associated with hearing loss and for co-occurring or new onset conditions due to hearing loss.

Although health-related and financial consequences of undetected and untreated hearing loss are expected to be significant, hearing screening is not a routine component of primary care. The United States (US) Preventative Services Task Force acknowledges the importance of identification and treatment of hearing loss for older adults but states that there is inadequate evidence to determine the balance of benefit and harm of screening for hearing loss in asymptomatic adults age 50 years and older [13]. Despite the lack of clinical trial evidence, the Department of Health and Human Services, American Speech-Language-Hearing Association, National Academies of Sciences, Engineering and Medicine (NASEM) and systematic reviews, recommend that older adults be screened for hearing loss [14–16].

Conflicting recommendations may explain below optimal hearing screening (HS) rates for older adults. Only 20% of physicians routinely screen for hearing loss and only 14% of adults 65–74 years and 16% of those 75+ years are screened [17, 18]. Other factors influencing whether or not older adults complete a HS include individual and familial attitudes toward hearing loss, perceived social pressure, awareness of HS options, perceived expense of treatment and hearing aids, primary care provider (PCP) dismissal of hearing impairment concerns, and inadequate PCP reimbursement [13, 19–25]. Primary care practices may also be limited on space to reserve a quiet room for testing or limited on time to add an additional screen into an already busy workflow. Improving HS uptake requires consideration of factors at the patient, family, provider, practice, community and societal levels.

Unfortunately, those who complete and fail a HS may not seek help for it, influenced by perceptions and attitudes of hearing loss and disability [19–21, 26]. Some studies have shown more than 60% of adults who failed an online HS followed up at an audiology clinic [27]. Ideally, those who fail a HS should undergo diagnostic testing by an audiologist to confirm and characterize the loss and develop a plan for accommodations or treatment. For certain losses, hearing aids may be an option for intervention. From 1977 to 2016, the US Food and Drug Administration (FDA) required that prospective adult hearing aid users should have a medical evaluation by a licensed physician to determine the presence of any of the eight ‘red flag’ conditions (those that may require further medical consultation and/or intervention; Table 1) or sign a waiver for that evaluation. This FDA requirement for medical evaluation for red flag conditions was not evidence based. In December 2016, the FDA eliminated the long-standing requirement for medical evaluation but continues to require hearing aid dispensers provide prospective users the opportunity to review information about possible ‘red flags’ prior to the sale of a hearing aid should users decide to pursue medical consultation. Self-identification of medical contraindications becomes essential. Yet, no study has ever evaluated whether assessment by the patient (self-assessment), PCP, or a diagnostic audiologist could accurately identify these conditions, as compared with an expert medical opinion by an otolaryngology provider.

**Table 1:** Eight Red Flag Conditions

(i) Visible congenital or traumatic deformity of the ear;	
(ii) History of active drainage from the ear within the previous 90 days;	
(iii) History of sudden or rapidly progressive hearing loss within the previous 90 days;	
(iv) Acute or chronic dizziness;	
(v) Unilateral hearing loss of sudden or recent onset within the previous 90 days;	
(vi) Audiometric air-bone gap equal to or greater than 15 dB (dB) at 500 hertz (Hz), 1000 Hz, and 2000 Hz;	
(vii) Visible evidence of significant cerumen accumulation or a foreign body in the ear canal;	
(viii) Pain or discomfort in the ear.	

### Study aims

To address these challenges to hearing health care, we are conducting an implementation-effectiveness study aimed to (a) evaluate older adults’ uptake of HS and follow-up for audiologic testing if indicated, and (b) determine the accuracy of assessment and need for evaluation of ‘red flag’ conditions. We proposed to explore the patient, family and provider attitudes toward hearing loss, screening and medical care, and estimate the cost-benefit of three HS strategies in primary care practices. The three HS strategies for comparison include: (1) printed educational materials about hearing loss and access to at-home telephone-based HS (tele-HS), (2) PCP encouragement for HS plus printed materials and access to at-home tele-HS, and (3) PCP encouragement for HS plus printed materials and in-clinic tele-HS. Our primary hypothesis is that education and active encouragement by the PCP to perform and follow through on the screening in the office (Group 3) will result in higher HS rates than education, encouragement and instructions for home HS testing (Group 2) or education and instructions for home HS testing (Group 1). This pragmatic study intends to generate real-world evidence. Our collaboration with both a primary care practice-based research network and a NIH-funded nationwide network of community and academic otolaryngology practices is an effort to reduce the time lag from evidence to implementation and spread.

## Methods

### Study design overview

Our goal is to develop evidence that can inform hearing health care for adults ages 65 to 75 years. In this implementation-effectiveness hybrid type 1 study (testing effects of the intervention while gathering data on implementation) we will determine the level of PCP involvement needed to inform and encourage older adults to follow through with routine HS and determine if providing the opportunity to complete the tele-HS in-clinic as part of the primary care appointment versus access to tele-HS at home increases HS completion. We study the costs associated with three different HS strategies and the patient, family and provider attitudes toward hearing loss and treatment. An implementation-effectiveness hybrid type 1 study as described here is best fit for examining HS in routine primary care because there is strong face validity for clinical intervention, minimal risk, and supporting evidence but not yet consensus for national HS recommendations [28]. Our evaluation of patients, PCPs and audiologists reliably assessing ‘red flag’ conditions further contributes valuable information for future implementation studies to scale-up HS and patient education for managing hearing loss.

### Site recruitment and context

Primary care practices for this study’s primary care cohort are from the 25 Family Medicine and Internal Medicine practices in the Duke Primary Care Research Consortium (PCRC). An initial query of data in our electronic data warehouse (DEDUCE) provide estimates per clinic for the number of unique patients age 65–75 seen between 8/1/15–7/30/16. Our site recruitment plan includes invitations to 8 of the 11 clinics with the highest numbers of potentially eligible patients (3 Family Medicine, 5 Internal Medicine); of these, 6 clinics will participate in the study with 2 clinics per intervention group. Each group is balanced based on three factors: primary care specialty (Family Medicine or Internal Medicine), practice size (number of providers and eligible patients), and ability/willingness to implement the intervention.

Our study additionally includes otolaryngology head and neck surgery (OHNS) clinics in order to address our aim on accuracy of assessment of medical conditions and those which require evaluation. In addition to the local OHNS clinic that supports the patients seen in the PCP cohort, we will select at least ten OHNS clinics from Creating Healthcare Excellence through Education and Research (CHEER) Practice-based Research Network for participation in our study, with Duke serving as the Central IRB. CHEER is a multi-site national research network of physicians and allied health professionals specializing in disorders of the ear, nose and throat funded initially (in the 10 year start-up phase) by the NIH/NIDCD. OHNS clinics in the CHEER network are fully trained in regulatory and data quality standards, have together developed standard operating procedures, and have a multi-level governance structure that supports rapid study start up and patient recruitment [29].

### Patient inclusion and exclusion criteria

**Fig. 1:**
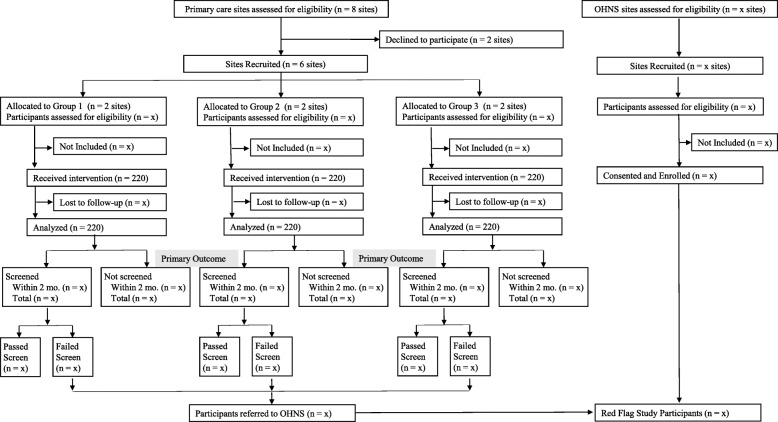
Consort Diagram of Study Patients. Abbreviations:OHNS = Otolaryngology Head and Neck Surgery

The total sample size for the PCP cohort is 660 study patients (220 per intervention group, 110 per clinic). The proposed sample to assess red flags in the OHNS multi-site CHEER research network is 500 patients. A CONSORT diagram of patient flow in the study is depicted in Fig. 1.

Inclusion criteria are:

- 65–75 years of age
- Being seen for non-acute follow-up or annual appointment for primary care

Exclusion criteria for this study are as follows:

- Current or prior history of hearing aid use
- Prior evaluation by an audiologist in the past 5 years and/or self-reports a hearing loss diagnosis
- Being seen for an acute illness or hospitalization

### Sample size

Estimates of sample size for HS and audiology/OHNS follow up were determined as follows. Patients in Group 3 (in-office testing) may refuse to participate in the screening. Thus, although participation in that group is expected to be high, it will likely not equal 100%. Rates of Tele-HS completion are estimated at: 25% for Group 1, 50% for Group 2 and 80% for Group 3. We estimate that no more than a total of 100 patients will fail HS and be referred on for further testing (based on estimated total *N* = 600 for those patients eligible for analysis). The rationale for this is as follows: If 80% of patients in Group 3 undergo HS, we expect 25% may fail (*N* = 40); for Group 2 if 50% take the test, 25% or *N* = 25 will fail; for Group 1, we have hypothesized that a higher percentage of test takers may suspect hearing loss, and for this reason the failure rate may be higher than 25%. We estimate that 25% will take the HS test, and as many as half may fail, or *N* = 25. Thus, 40 + 25 + 25 = 90, and considered up to 100 might follow up for audiological diagnostic testing from the PCP cohort. If rates of test completion in the three groups are as expected (25, 50, 80%), then, using standard methods for the calculation of power [30], power is greater than 99% to declare significance for the primary contrast of interest (Group 3 vs. Groups 1 and 2) assuming alpha = 0.05, two tailed, *n* = 600, 200 per group. Indeed, if the rate is 80% as expected for Group 3, power will equal 80% to detect differences in rates of test completion, if the rate in Groups 1 and 2 is as low as 30%.

We proposed 500 patients will be recruited for the medical evaluation aim and determined feasibility of enrollment using a de-identified, patient level database from the CHEER Network. The Retrospective Data Collection database is based on 1 year of data and includes data from 22 of 30 sites. Using this conservative subgroup of the network, the database was filtered for patients age 65–75 with a diagnosis of hearing loss. This yielded a total of 11,414 unique patients. Further, filtering all but 4 sites that are likely participants in the study (based on volumes) yielded 3424 unique patients in 1 year, confirming that the proposed sample size of 500 recruited over 3 years is feasible.

### Patient recruitment and informed consent

Duke University serves as the study coordinating center for all research activities and is responsible for communicating across all sites and patients. Study approval from the Duke University Institutional Review Board (IRB) includes a waiver of consent due to participation having minimal risk and that it would not adversely affect individual welfare or patient rights. Study information provided to participating patients includes all elements of informed consent; however, written or verbal consent are not obtained or required. Personal identifiers are not stored with study data to protect confidentiality of patient during and after the trial. The pragmatic study design is dependent on preserving clinical flow. Obtaining written consent is not practical due to the large sample size per primary care practice.

A total of 955 subjects will be screened for eligibility to enroll 660 patients for the PCP aim. These 660 patients will be across six sites, 110 per practice in each of the six participating practice sites, two practices per intervention arm (*N* = 220 patients per group). Prospective enrollment is scheduled at each site based on the number of patients age 65–75 years, and participating provider schedules on a given day, until a total of 110 patients are enrolled. A member of the clinical care team first introduces the study to prospective patients. Either a member of the clinical care team or the study coordinator then gives the study informational consent page along with the self-screening study eligibility form to the patient. Patients have the option to participate; completing the study eligibility form implies patient consent. Patients who meet eligibility based on responses in the form are considered enrolled and receive a study packet appropriate for the intervention group to which that practice site is assigned.

A log is used to track the number of patients in the PCP practice who meet eligibility criteria but decline participation, and reasons for not participating in the study. Demographics (sex, age, race/ethnicity) of all screened patients are recorded. No compensation is provided for study participation as all activities are considered standard of care. The hearing screening is provided free of charge, whether performed in the office or at home. All data collected clinically are managed electronically centrally at Duke University. Patients with a HS failure are referred for audiological assessment as described below.

### Hearing screening intervention groups

Eligible and enrolled patients in all groups receive printed information about hearing loss, a questionnaire packet, and tele-HS specifics for their intervention group. The three tele-HS intervention groups are depicted in Table 2 with PCP time, involvement and resources increasing from Group 1 to 2 to 3. Group 3 has the most office-based involvement with the tele-HS in a quiet room onsite rather than receiving instructions to complete it at home. Patients in Groups 1 and 2 have 2 months to follow through with the instructions to complete the tele-HS at home.

**Table 2:** Strategies Used for Each Intervention Group

Strategies	Group 1	Group 2	Group 3
Telephone-based Hearing Screening			
Receives instructions to take test from home	X	X	
Offered in-office during the visit			X
PCP Encouragement/Counsel on Hearing Screening Importance			
PCP encouragement during office visit		X	X
Educational Materials			
Written information on warning signs of hearing loss, consequences of hearing loss, and possible interventions	X	X	X

Although several HS methods are available and in use [31], options vary by cost and maintenance of instrumentation, time or resource intensity, and sensitivity and specificity. The tele-HS selected for this study is a publicly available telephone-based triple-digit HS test, modified for use in the US [32, 33], and utilizes a speech-in-noise test combined with simple keypad subject response. The speech-in-noise test is thought to be less prone to inconsistencies due to background noise and instrumentation requirements than other screening tests. These features, combined with reported good sensitivity and specificity (0.80 and 0.83 respectively, using pure tone average > 20 dB HL as the criterion measure) make this an ideal HS test administered in the patient’s home or in the PCP office.

Group 3 in-office use of the tele-HS is completed on a ‘land line’ telephone. Patient information packets for Groups 1 and 2 instruct patients to complete the HS at home from a land line (versus mobile device or cell phone) in their own home or that of a family member or friend. Patients who conduct the tele-HS receive results at the end of the call (pass or fail with formal testing by a specialist recommended), either in the office (Group 3) or home (Groups 1 and 2).

### Failed HS and referred

Failure rate of those completing the tele-HS test are tracked but this is not considered an outcome of the study. A study coordinator contacts patients who fail the tele-HS either in the PCP office or by phone and asks patients to follow up with an appointment for diagnostic audiological testing and medical evaluation with a specialty provider at the Duke Head and Neck Surgery and Communication Disorders Clinic. The follow-up visit is described as part of routine care for hearing loss and it is recommend to be completed within 4 months following the tele-HS failure. The number of patients who schedule and who attend the follow-up visit are documented.

### Red flags

PCPs complete a questionnaire during their evaluation of the patient to assess for ‘red flag’ conditions during the index office visit. While in the audiologist’s office, and prior to audiological testing, patients who failed the tele-HS complete a short questionnaire to indicate their awareness of whether they have any of the ‘red flag’ conditions. The patient self-assessment of medical contraindications is especially important with the 2016 change by the FDA no longer requiring medical evaluation for red flag conditions. Hearing aid dispensers rely on prospective users to review information to make an informed decision on the purchase of a hearing aid but patient/purchaser awareness of risk is unclear.

Audiological diagnostic assessment for patients who failed the tele-HS is performed in a sound-treated booth utilizing earphones. It includes pure tone audiometry for air and bone conduction (audiogram), speech testing, and tympanometry, per standard of care for hearing diagnostic testing. Audiologists document their findings, including whether or not a red flag condition or other significant non-red flag condition is present and should be considered a medical contraindication to hearing aid fitting.

Of the eight red flag conditions, cerumen impaction is expected to be present in up to 30% of older adults with hearing loss [34]. In order to assess the potential impact of this condition on hearing aid fitting, an audiologist in the specialty clinic performs an otoscopic exam, documents findings, and performs an audiogram (whether or not cerumen was identified in otoscopic exam). If cerumen impaction is identified, the medical specialist performs microscopic cleaning, and patients are sent back to the audiologist for a repeat audiogram for the affected ear only. This provides data on the impact of cerumen impaction on pure-tone thresholds. Results of the audiological and medical evaluation are discussed with the patient by the medical provider, and if appropriate, additional educational materials on hearing loss and suggested interventions are provided. Any identified medical conditions are managed as per standard of care.

**Fig. 2:**
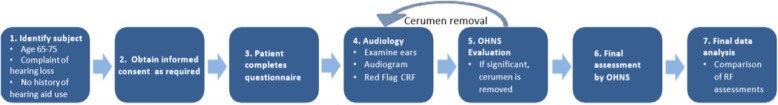
CHEER Red Flag Study Flow. Abbreviations: CRF = Case report form, OHNS = Otolaryngology Head and Neck Surgery, RF = Red flag

Evaluation of red flag conditions on hearing and implications for hearing aid fitting are being studied with participation of patients in the PCP study and also with patients recruited from OHNS clinics in the CHEER network across the US. This approach of expanding enrollment from the CHEER network clinics is essential for increasing the likelihood of occurrence of medical conditions that might contraindicate hearing aid fitting. Procedures similar to the main study will be followed with at least 10 participating sites in the CHEER. The proposed sample is 500 adults (including those patients in the PCP cohort referred for evaluation after HS failure) ages 65–75 years who present for evaluation of hearing loss, either as a new patient or at follow-up, and do not currently or in the past use a hearing aid. A CHEER site study coordinator approaches patients who meet enrollment criteria upon clinic check-in, informs them about the study, the purpose and any time requirements, and obtains informational consent. Tele-HS will not be conducted. CHEER site studies then followed steps 2–7 as depicted in Fig. 2.

In order to approximate ‘real world’ audiology expertise in community-based and academic Otolaryngology Head and Neck Surgery clinics, we do not offer training or skills verification for the red flag study. We acknowledge that this subset of audiologists is not representative of all dispensing audiologists or hearing aid dispensers, and that findings are not imminently generalizable. However, this study and its design sought to provide important preliminary data for future research with a wider population of providers.

Printed materials on study procedures are provided as guidance and to ensure documentation standardization across sites (frequencies tested, recorded vs. live voice speech testing, equipment calibration, etc.). Per patient site payments are offered to offset the costs of repeat audiograms in the case of cerumen impaction. Repeat audiograms are not standard of care and sites would otherwise have sent patients to otolaryngology providers without performing audiometry, have cerumen removed, and then return for testing. No compensation is provided to patients as the burden of participation is low.

### Primary care practice-based HS intervention development

The PCRC Research Advisory Board will review our proposal and advise on the study procedures, budget, and approach to recruitment. Practice managers and PCPs work closely with the study team to design the tools and standard operating procedures compatible with the workflow in the clinic and to optimize provider involvement and patient recruitment and participation. Protocol training is provided at each site with additional 1:1 training for clinic staff prior to patient recruitment. No specific training in performing an ear exam is provided unless requested. Evaluation is to reflect the exam of a general practitioner. Each intervention arm is piloted with 15 patients in three of the six participating primary care practices (five patients per site, one site per intervention arm). Using operations management expertise, we gather data and feedback on implementation of encouragement protocols, medical evaluation of the ears, the administration of the HS, data collection in the office setting, and office flow to inform refinement of the protocol to assist in implementation.

### Effectiveness outcomes

The primary outcome measure is the percent of patients who complete the tele-HS within 2 months of their PCP visit. Although in-office tele-HS may have a higher compliance rate than at-home tele-HS, the failure rate may be greater among those who proceed with in-home HS as their compliance to complete the HS at home could be motivated by suspected hearing loss. Our secondary effectiveness outcomes measured within 4 months of the failed tele-HS include the:

- percent of patients who schedule follow-up diagnostic testing,
- percent of patients with an identify hearing loss with the tele-HS who attend the specialty visit for follow-up diagnostic audiologic testing,
- percent of patients with an identified hearing loss with the tele-HS who completed a plan for appropriate hearing loss intervention if indicated, and
- accuracy of assessment for red flag conditions comparing PCP and audiologist evaluations versus an otolaryngology provider evaluation (gold standard).

We will also explore the association of cerumen impaction with pure-tone thresholds.

### Barriers to implementation

#### Attitudes and perceptions

Data are sought to describe the attitudes of patients and the perceived attitudes of communication partners regarding hearing loss. PCPs record patients’ subjective assessment of the presence or absence of significant hearing impairment. All patients in primary care are asked to complete two questionnaires either during the index office visit or at home, returning them with a pre-addressed and stamped envelope. The Attitudes towards Loss of Hearing Questionnaire for Non-Hearing Aid Users (ALHQ) is a 22-item instrument that measures attitudes on five subdomains: denial of hearing loss, negative associations, negative coping strategies, manual dexterity and vision, and hearing-related esteem [35–37]. Each item is scored by the patient on a 5-point Likert scale indicating level of agreement (strongly disagree, neither agree nor disagree, to strongly agree). The Significant Other Scale for Hearing Disability (SOS-HEAR) questionnaire measures the perceived attitudes of communication partners regarding hearing loss [38]. Patients complete the 27 items based on their partner’s perspective, responding to each item on a 5-point Likert scale for no problems (=0) to a complete problem (=4). The instrument is scored to assess the effects of hearing impairment on the significant other in the following domains: changes to communication, communication burden, relationship changes, going out and socializing, emotional reactions to adaptations, and concern for the partner. Attitudes are explored for their association with willingness to pursue HS, diagnostic testing, and consideration of intervention.

Sixteen focus groups are planned to collect data that will to provide context to the questionnaire results from the main study. These groups will include 128–160 patients, communication partners, clinic staff and healthcare clinicians across study groups and clinical sites. Discussions aim to understand attitudes and perceptions that may support or impede pursuit of hearing health care.

#### Cognitive function

PCPs are asked to assess cognitive function and comment on functionality using a 3-point scale (normal, mildly impaired, moderately-severely impaired). We will examine if successful completion of the tele-HS is possible for those with mild or moderately-severe cognitive impairment.

#### Costs of primary care intervention

Direct costs for each primary care intervention group and the individual strategies are calculated with costs of the educational material, tele-HS, and estimated cost of PCP time to educate and encourage the patient using a guided script (designed to be less than 3 min). Indirect costs for in-clinic HS include cost of the space used and staff time to direct patients to the room for the tele-HS.

### Statistical considerations

The primary analysis is based on the anticipated 660 patients who are deemed eligible and receive a study packet in the main study (R33 phase). In each of six practices, it is expected that 110 patients will receive a study packet, with two practices per intervention arm (*N* = 220 patients in each group). Due to the anticipated high rate of screen failures and withdrawals, we plan to approach up to 2000 patients (completed self-screening study eligibility forms) to meet the goal of 660 study packets distributed to eligible patients.

The primary outcome measure is the proportion of patients in each group who dial the phone number and complete the test within 2 months of their PCP visit. To test efficacy, Groups 1 and 2 will be compared to Group 3. Patients who complete the tele-HS outside of the 2-month window are not included in this primary analysis but are included in the per protocol analysis.

Secondary outcome measures will assess if there are group differences in the proportion of patients in all groups who: a) schedule, b) complete the visit for diagnostic audiologic testing, and subsequently, c) complete a plan for appropriate hearing loss intervention, if indicated, within 4 months of initial hearing screening.

#### Analysis for the primary outcome

The difference in the Group proportions in test completion will be examined for the primary outcome by a test of proportions. If there is an overall difference in rates of test completion, follow-up pairwise contrasts will be conducted to assess which groups differ significantly, comparing Group 3 vs. Groups 1 and 2, and, secondly, Groups 1 vs 2. Although every effort will be made to balance practice populations, we realize that practices may differ in the attributes of recruited patients. To control for baseline group differences, in follow-up sensitivity tests, we will assess if the differences in site, demographic, and comorbid conditions impact the observed group differences by multinomial logistic regression.

## Discussion

This study is designed to answer real-world questions in hearing health care: what primary care model is best for motivating adults ages 65–75 years to complete a tele-HS and follow-up for medical evaluation and care if warranted, and how accurate are assessments of red flag conditions from patient, PCP and audiologists? We further assess barriers to implementation including attitudes, perceptions, cognitive impairment and costs in order to guide future implementation research that could test discrete implementation strategies [39]. We designed this study to begin with patients’ subjective assessment of the presence or absence of significant hearing loss in order to consider necessary education and behavior change strategies that could be tested for inclusion in the context of routine care. The burden of hearing loss at the individual, familial, healthcare, community and societal levels is immense, and increasing as the US population of older adults increases; yet, the population and public health impact is currently underestimated because too few people are screened, referred and treated.

NASEM convened an expert committee to study and provide recommendations in 2016 for improving the affordability and accessibility of hearing health care for adults in the US. Institutional, technological and regulatory changes are recommended [16]. Beyond hearing aids and assistive technologies, the committee importantly acknowledged that assessment of hearing difficulties and loss, diagnosis of underlying conditions, treatment and counseling are often overlooked. Our study addresses this gap directly by testing different approaches as part of routine primary care and measuring the percent of patients who follow recommendations as they transition in care to meet their needs. Factors associated with refusal or discontinuation of care will be informative for guiding uptake and future implementation efforts.

This study will establish evidence for conditions that should be contraindicated for hearing aid fitting and the impact of the most common red flag condition, cerumen, on hearing thresholds. Although the FDA has eliminated the requirement for medical evaluation of red flag conditions as it was in place without evidence to support it, we are still without research on these conditions as contraindications. The ‘red flag’ requirement for education of potential hearing aid users remains in place to ensure users are aware that there may be a medically treatable cause of their hearing loss. Our findings will help inform both practice and policies for audiologists, hearing aid dispensers and labeling of amplification devices.

As part of our implementation-effectiveness design, we chose not to randomize the patients or the sites for pragmatic reasons. Patient randomization to the intervention would mean that all clinics would have to be able to employ each screening strategy. In a real-world scenario, clinics will likely implement only one screening strategy. Clinic randomization is also not performed due to budget limitations for involving a high number of clinics and an unknown intra-class correlation for an adequately-powered cluster randomized trial, which would require at least 10 clinics per intervention arm. Site-level implementation allows us to train providers and measure costs of implementing a new approach as part of standard of care for this patient population. A site-level design increases pragmatism, feasibility of PCP engagement, and simplifies the patient recruitment process in comparison with a patient-level or patient-randomized design. We aim to achieve balance in site-level characteristics (PCP specialty, number of providers and eligible patients) and the number of patients is held constant across sites and interventions arms. However, we will plan to evaluate for imbalance in patient characteristics (race/ethnicity, sex) of study participants and non-participants between groups, and demographics of the study sample (approached, declined, enrolled) to the overall practice demographics for this age group.

In conclusion, our study addresses critical gaps in hearing health care. If successful, we will identify a primary care-based approach that increases uptake of tele-HS and medical follow-up. The design of our study will accelerate translation into practice and establish the foundation for widespread large implementation and future research to reach medically underserved populations. Hearing is vital to daily function and participation at home, in the workplace and in the community; it is a public health imperative that research continues to advance access and accessibility to hearing health care services and technologies.

## Data Availability

Data sharing is not applicable to this article as no datasets were generated or analyzed during the current study. Final trial data will be accessible in accordance with rules from the funding agency.

## Abbreviations

ALHQ: Attitudes towards Loss of Hearing Questionnaire for Non- Hearing Aid Users
CHEER: Creating Healthcare Excellence through Education and Research
CRF: Case report form (Fig. 2 only)
dB: Decibel
FDA: Food and Drug Administration
HS: Hearing screening
Hz: Hertz
IRB: Institutional review board
NIH/NIDCD: National Institutes of Health / National Institute on Deafness and Other Communication Disorders
OHNS: Otolaryngology Head and Neck Surgery Clinic
PCP: Primary care provider
PCRC: Primary Care Research Consortium
RF: Red flag (Fig. 2 only)
SOS-HEAR: Significant other Scale for Hearing Disability
Tele-HS: Telephone-based hearing screen
US: United States
